# Supplementary material for: Two neuropeptides that promote blood feeding in Anopheles stephensi mosquitoes
Source: eLife. 2026 May 19;14:RP108625. doi: 10.7554/eLife.108625 (PMC13186566; doi:10.7554/eLife.108625)
Supplement: Supplementary file 1. [file elife-108625-supp1.pdf]

GLMM analysis of feeding behaviours in *An. stephensi* females (figure 1D, E)

Data from D0 (day of emergence) were excluded from the GLMM analysis because newly emerged females show no feeding behaviour on this day, resulting in no variance in the response variable to model.

Model 1: Whether the experimental groups differ in blood-feeding behaviour across age?

Random variables: BioID (replicate identity), larval.batch (larval batch)

Fixed variables: Day(age), Group (co-housed vs virgin)

```
model1 <- glmer(Bloodfed_binary ~ Day * Group + (1|BioID) + (1|larval.batch),
        data = mosquito_data_filtered,
        family = binomial)
```

Model summary:

Characteristic	Odds	Probability (%)	OR[1]	95% CI	p-value
**Day**					
D1	1.41	58.5	—	—	
D2	2.67	72.8	1.90**	1.29, 2.81	**0.001**
D3	4.87	83.0	3.47***	2.25, 5.35	**<0.001**
D4	6.35	86.4	4.52***	2.86, 7.13	**<0.001**
D5	4.64	82.3	3.30***	2.15, 5.08	**<0.001**
**Group**					
co-housed	1.41	58.5	—	—	
virgin	1.20	54.5	0.86	0.59, 1.23	0.403
**Day * Group**					
D2 * virgin	2.44	70.9	1.74	0.99, 3.05	0.053
D3 * virgin	0.95	48.7	0.68	0.38, 1.21	0.189
D4 * virgin	0.56	35.9	0.40**	0.22, 0.72	**0.002**
D5 * virgin	0.90	47.4	0.64	0.36, 1.14	0.130

[1] *p<0.05; **p<0.01; ***p<0.001

Abbreviations: CI = Confidence Interval, OR = Odds Ratio

Post-hoc analysis via estimated marginal means (EM means)

1) Age-effects within each group (p-value adjustment: Tukey's method)

Group = co-housed:

contrast	estimate	SE	df	z.ratio	p.value
D1 - D2	-0.6423	0.199	Inf	-3.234	0.0107
D1 - D3	-1.2432	0.221	Inf	-5.614	<.0001
D1 - D4	-1.5085	0.233	Inf	-6.479	<.0001
D1 - D5	-1.1946	0.220	Inf	-5.429	<.0001
D2 - D3	-0.6009	0.228	Inf	-2.638	0.0637
D2 - D4	-0.8662	0.242	Inf	-3.577	0.0032
D2 - D5	-0.5523	0.229	Inf	-2.416	0.1111
D3 - D4	-0.2653	0.261	Inf	-1.018	0.8473
D3 - D5	0.0485	0.248	Inf	0.196	0.9997
D4 - D5	0.3138	0.262	Inf	1.199	0.7521

Group = virgin:

contrast	estimate	SE	df	z.ratio	p.value
D1 - D2	-1.1958	0.210	Inf	-5.697	<.0001
D1 - D3	-0.8564	0.201	Inf	-4.262	0.0002
D1 - D4	-0.5857	0.192	Inf	-3.050	0.0194
D1 - D5	-0.7529	0.199	Inf	-3.791	0.0014
D2 - D3	0.3394	0.220	Inf	1.542	0.5353
D2 - D4	0.6100	0.216	Inf	2.826	0.0379
D2 - D5	0.4429	0.221	Inf	2.007	0.2625
D3 - D4	0.2706	0.207	Inf	1.308	0.6859
D3 - D5	0.1035	0.211	Inf	0.491	0.9882
D4 - D5	-0.1671	0.207	Inf	-0.806	0.9288

Results are given on the log odds ratio (not the response) scale.
P value adjustment: tukey method for comparing a family of 5 estimates

2) Pair-wise comparison at each day (p-value adjustment: Bonferroni method)

```
Day = D1:
 contrast              estimate    SE  df z.ratio p.value
 (co-housed) - virgin    0.156 0.186 Inf   0.836  0.4034

Day = D2:
 contrast              estimate    SE  df z.ratio p.value
 (co-housed) - virgin   -0.398 0.217 Inf  -1.830  0.0672

Day = D3:
 contrast              estimate    SE  df z.ratio p.value
 (co-housed) - virgin    0.542 0.228 Inf   2.376  0.0175

Day = D4:
 contrast              estimate    SE  df z.ratio p.value
 (co-housed) - virgin    1.078 0.236 Inf   4.570  <.0001

Day = D5:
 contrast              estimate    SE  df z.ratio p.value
 (co-housed) - virgin    0.597 0.225 Inf   2.656  0.0079

Results are given on the log odds ratio (not the response) scale.
```

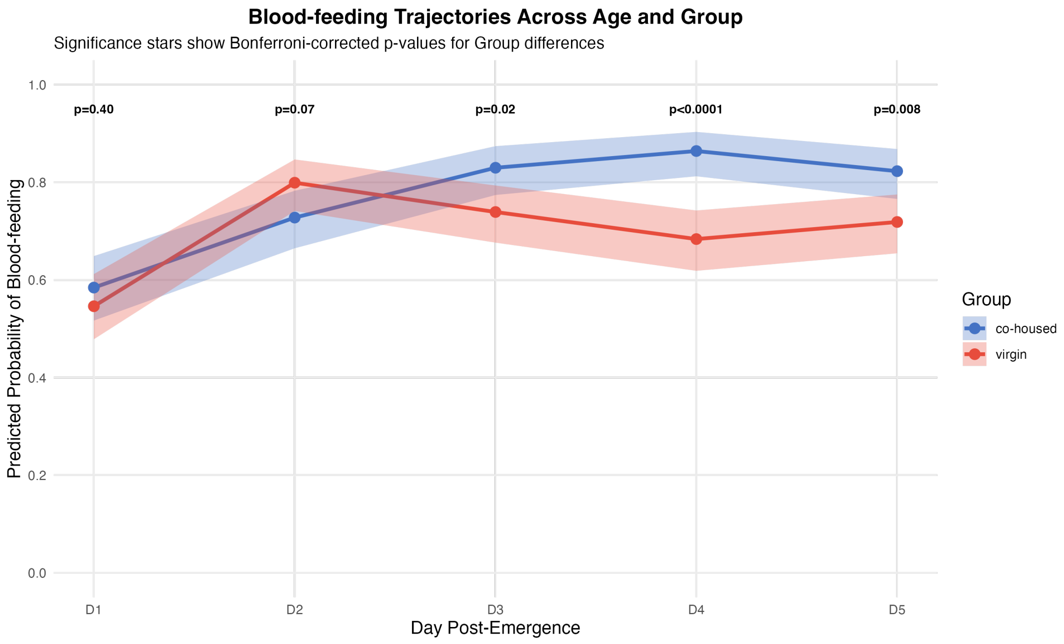

<u>Conclusions:</u> Blood-feeding probability of co-housed females increases with age. This trend is weaker in virgins.

Mated females feed significantly higher than virgins as they age (D3-D5), the differences being most pronounced on D4.

Model 2: Effect of mating on the blood-feeding behaviour of co-housed females

Random variables: BioID (replicate identity)

Fixed variables: Day(age), Mating status (yes or no)

```
model2 <- glmer(Bloodfed_binary ~ Day * Mating + (1|BioID),
            data = mated_only,
            family = binomial)
```

Model summary:

Characteristic	Odds	Probability (%)	OR[1]	95% CI	p-value
**Day**					
D1	1.37	57.8	—	—	
D2	2.14	68.2	1.56	1.00, 2.46	0.052
D3	2.23	69.0	1.63	0.87, 3.06	0.130
D4	4.23	80.9	3.09*	1.21, 7.88	**0.018**
D5	1.54	60.6	1.12	0.18, 6.89	0.902
**Mating**					
n			—	—	
y	2.63	72.5	1.92	0.49, 7.46	0.347
**Day * Mating**					
D2 * y	1.22	55.0	0.89	0.20, 3.92	0.878
D3 * y	2.18	68.6	1.59	0.34, 7.42	0.555
D4 * y	1.14	53.3	0.83	0.16, 4.42	0.827
D5 * y	2.28	69.5	1.67	0.17, 16.2	0.660

[1] *p<0.05; **p<0.01; ***p<0.001

Abbreviations: CI = Confidence Interval, OR = Odds Ratio

Post-hoc analysis via estimated marginal means (EM means)

Effect of mating (y) on each day (p-value adjustment: Bonferroni method)

```
Day = D1:
 contrast estimate    SE  df z.ratio p.value
 y - n       0.651 0.693 Inf   0.939  0.3475

Day = D2:
 contrast estimate    SE  df z.ratio p.value
 y - n       0.535 0.301 Inf   1.777  0.0756

Day = D3:
 contrast estimate    SE  df z.ratio p.value
 y - n       1.115 0.368 Inf   3.027  0.0025

Day = D4:
 contrast estimate    SE  df z.ratio p.value
 y - n       0.464 0.504 Inf   0.920  0.3575

Day = D5:
 contrast estimate    SE  df z.ratio p.value
 y - n       1.162 0.934 Inf   1.245  0.2133

Results are given on the log odds ratio (not the response) scale.
```

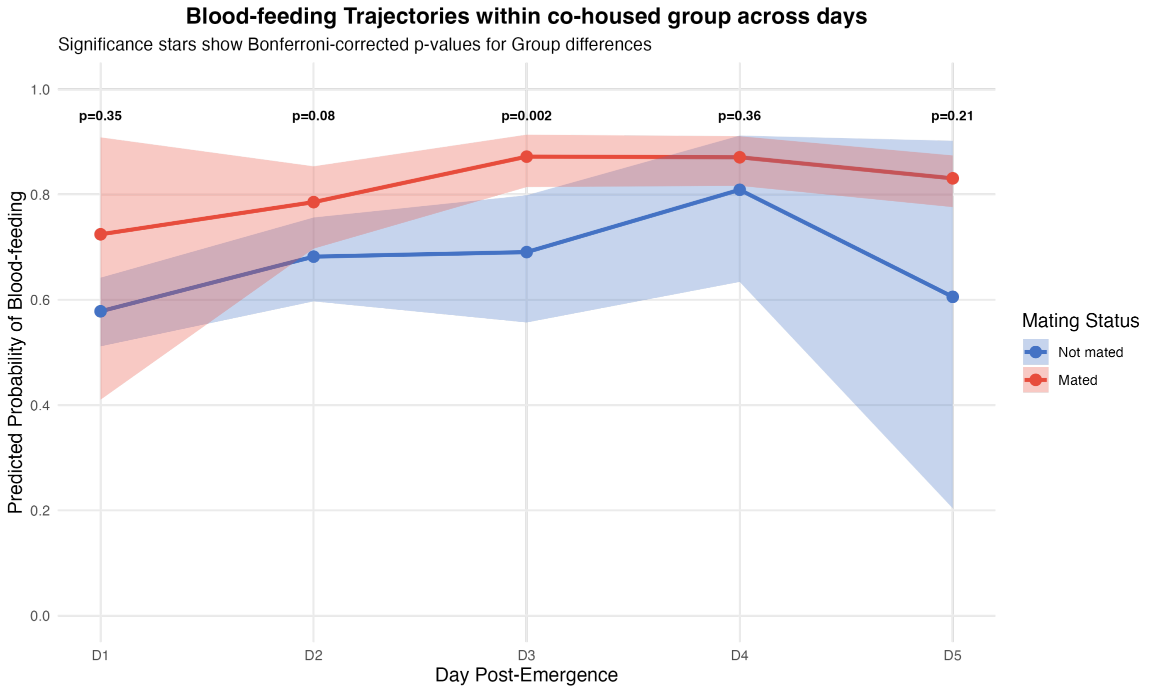

Conclusions: Within co-housed females, mating status shows only a brief, significant effect on blood-feeding on D3. The pronounced differences observed in the blood-feeding behaviour of virgin and co-housed females on Days 4-5 (model 1) appear to be mediated by male presence rather than mating per se, as mated and unmated females within the co-housed group show no significant differences during these time points.

<u>Model 3: Whether the experimental groups differ in their second-blood meal, across age?</u>

Random variables: BioID (replicate identity)

Fixed variables: Day(age), Group (co-housed vs virgin)

```
model3 <- glmer(Bloodfed_binary ~ Day * Group + (1|BioID),
          data = mosquito_data,
          family = binomial)
```

Model summary:

Characteristic	Odds	Probability (%)	OR[1]	95% CI	p-value
**Day**					
DB1	0.51	33.8	—	—	
DB2	0.21	17.4	0.41***	0.29, 0.60	**<0.001**
DB3	0.65	39.4	1.28	0.92, 1.78	0.143
DB4	0.40	28.6	0.78	0.55, 1.10	0.154
**Group**					
co-housed	0.51	33.8	—	—	
virgin	4.35	81.3	8.58***	5.81, 12.7	**<0.001**
**Day * Group**					
DB2 * virgin	1.47	59.5	2.90***	1.67, 5.02	**<0.001**
DB3 * virgin	0.47	32.0	0.92	0.54, 1.55	0.748
DB4 * virgin	0.97	49.2	1.91*	1.11, 3.28	**0.020**

[1] *p<0.05; **p<0.01; ***p<0.001

Abbreviations: CI = Confidence Interval, OR = Odds Ratio

(note: PBM abbreviated as “B”)

Post-hoc analysis via estimated marginal means (EM means)

1) Age-effects within each group (p-value adjustment: Tukey's method)

```
Group = co-housed:
 contrast  estimate    SE  df z.ratio p.value
 DB1 - DB2   0.8849 0.188 Inf   4.703  <.0001
 DB1 - DB3  -0.2477 0.169 Inf  -1.467  0.4579
 DB1 - DB4   0.2499 0.175 Inf   1.424  0.4841
 DB2 - DB3  -1.1326 0.174 Inf  -6.498  <.0001
 DB2 - DB4  -0.6350 0.180 Inf  -3.524  0.0024
 DB3 - DB4   0.4976 0.161 Inf   3.092  0.0107

Group = virgin:
 contrast  estimate    SE  df z.ratio p.value
 DB1 - DB2  -0.1790 0.210 Inf  -0.852  0.8296
 DB1 - DB3  -0.1619 0.210 Inf  -0.773  0.8667
 DB1 - DB4  -0.3952 0.217 Inf  -1.822  0.2629
 DB2 - DB3   0.0171 0.202 Inf   0.085  0.9998
 DB2 - DB4  -0.2162 0.210 Inf  -1.032  0.7308
 DB3 - DB4  -0.2332 0.209 Inf  -1.117  0.6792

Results are given on the log odds ratio (not the response) scale.
P value adjustment: tukey method for comparing a family of 4 estimates
```

2) Pair-wise comparison at each day (p-value adjustment: Bonferroni method)

```
Day = DB1:
 contrast              estimate    SE  df z.ratio p.value
 (co-housed) - virgin     -2.15 0.199 Inf -10.800  <.0001

Day = DB2:
 contrast              estimate    SE  df z.ratio p.value
 (co-housed) - virgin     -3.21 0.199 Inf -16.129  <.0001

Day = DB3:
 contrast              estimate    SE  df z.ratio p.value
 (co-housed) - virgin     -2.06 0.180 Inf -11.492  <.0001

Day = DB4:
 contrast              estimate    SE  df z.ratio p.value
 (co-housed) - virgin     -2.79 0.194 Inf -14.397  <.0001

Results are given on the log odds ratio (not the response) scale.
```

(note: PBM abbreviated as "B")

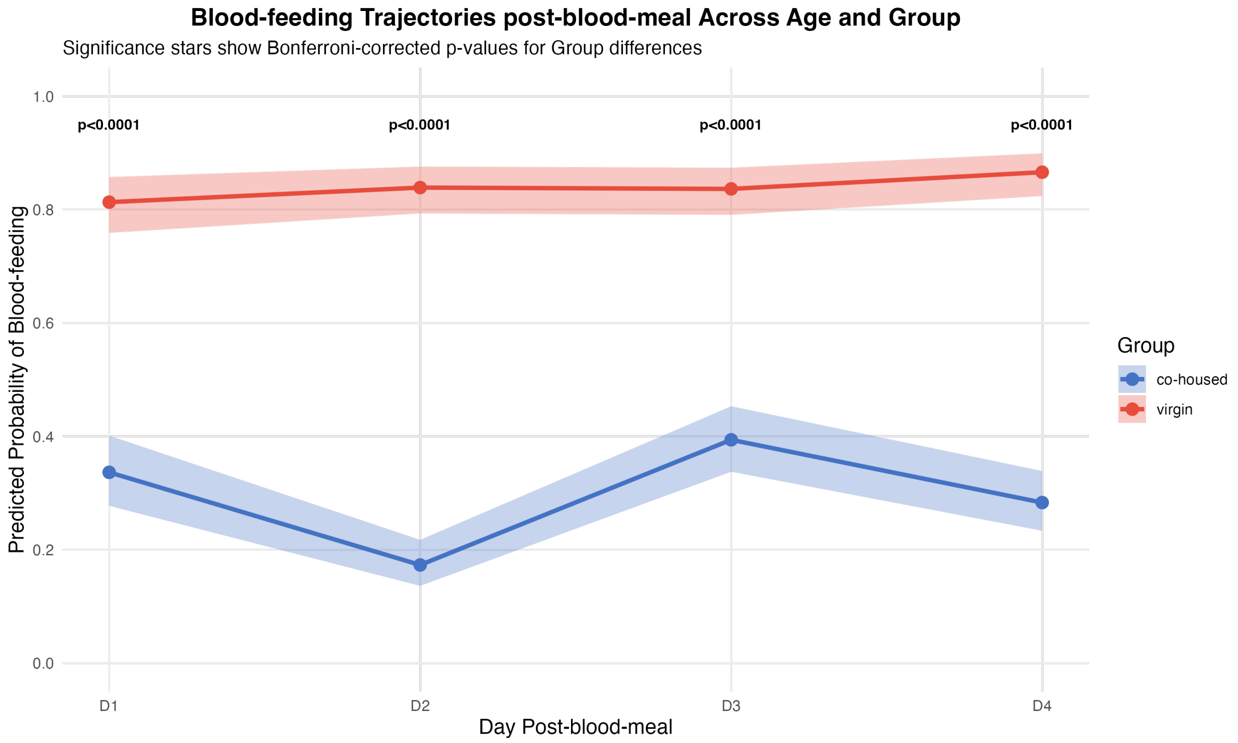

<u>Conclusions:</u> Co-housed and virgin females show markedly different appetites, post-blood meal. Co-housed females (assumed mated), show a strong sustained suppression, while virgins continue to feed with high propensity, at least up to 4 days after a replete blood meal.

Model 4: Whether the experimental groups differ in sugar-feeding behaviour across age?

Random variables: BioID (replicate identity)

Fixed variables: Day(age), Group (co-housed vs virgin)

```
model4 <- glmer(Sugar.fed_binary ~ Day * Group + (1|BioID),
        data = mosquito_data_filtered,
        family = binomial)
```

Model summary:

Characteristic	Odds	Probability (%)	OR[1]	95% CI	p-value
**Day**					
D1	0.41	29.1	—	—	
D2	12.37	92.5	30.1***	15.6, 57.9	**<0.001**
D3	17.88	94.7	43.4***	20.6, 91.8	**<0.001**
D4	33.53	97.1	81.5***	31.6, 210	**<0.001**
D5	28.03	96.6	68.1***	28.3, 164	**<0.001**
**Group**					
co-housed	0.41	29.1	—	—	
virgin	0.88	46.8	2.13***	1.38, 3.27	**<0.001**
**Day * Group**					
D2 * virgin	0.21	17.4	0.51	0.21, 1.23	0.134
D3 * virgin	0.09	8.3	0.21***	0.09, 0.52	**<0.001**
D4 * virgin	0.16	13.8	0.39	0.12, 1.30	0.126
D5 * virgin	0.15	13.0	0.37	0.12, 1.13	0.080

[1] *p<0.05; **p<0.01; ***p<0.001

Abbreviations: CI = Confidence Interval, OR = Odds Ratio

Post-hoc analysis via estimated marginal means (EM means)

1) Age-effects within each group (p-value adjustment: Tukey's method)

Group = co-housed:

contrast	estimate	SE	df	z.ratio	p.value
D1 - D2	-3.403	0.334	Inf	-10.185	<.0001
D1 - D3	-3.771	0.382	Inf	-9.884	<.0001
D1 - D4	-4.400	0.484	Inf	-9.093	<.0001
D1 - D5	-4.221	0.448	Inf	-9.421	<.0001
D2 - D3	-0.368	0.448	Inf	-0.823	0.9238
D2 - D4	-0.997	0.537	Inf	-1.855	0.3419
D2 - D5	-0.818	0.506	Inf	-1.617	0.4861
D3 - D4	-0.629	0.568	Inf	-1.106	0.8032
D3 - D5	-0.449	0.538	Inf	-0.835	0.9198
D4 - D5	0.179	0.615	Inf	0.292	0.9984

Group = virgin:

contrast	estimate	SE	df	z.ratio	p.value
D1 - D2	-2.737	0.296	Inf	-9.238	<.0001
D1 - D3	-2.224	0.256	Inf	-8.685	<.0001
D1 - D4	-3.454	0.387	Inf	-8.916	<.0001
D1 - D5	-3.225	0.354	Inf	-9.114	<.0001
D2 - D3	0.514	0.337	Inf	1.525	0.5461
D2 - D4	-0.717	0.445	Inf	-1.613	0.4887
D2 - D5	-0.488	0.416	Inf	-1.173	0.7668
D3 - D4	-1.231	0.419	Inf	-2.937	0.0274
D3 - D5	-1.001	0.388	Inf	-2.579	0.0743
D4 - D5	0.230	0.485	Inf	0.474	0.9897

Results are given on the log odds ratio (not the response) scale.
P value adjustment: tukey method for comparing a family of 5 estimates

2) Pair-wise comparison at each day (p-value adjustment: Bonferroni method)

Day = D1:

contrast	estimate	SE	df	z.ratio	p.value
(co-housed) - virgin	-0.7546	0.219	Inf	-3.441	0.0006

Day = D2:

contrast	estimate	SE	df	z.ratio	p.value
(co-housed) - virgin	-0.0887	0.392	Inf	-0.226	0.8210

Day = D3:

contrast	estimate	SE	df	z.ratio	p.value
(co-housed) - virgin	0.7932	0.407	Inf	1.950	0.0512

Day = D4:

contrast	estimate	SE	df	z.ratio	p.value
(co-housed) - virgin	0.1911	0.582	Inf	0.328	0.7426

Day = D5:

contrast	estimate	SE	df	z.ratio	p.value
(co-housed) - virgin	0.2413	0.529	Inf	0.456	0.6485

Results are given on the log odds ratio (not the response) scale.

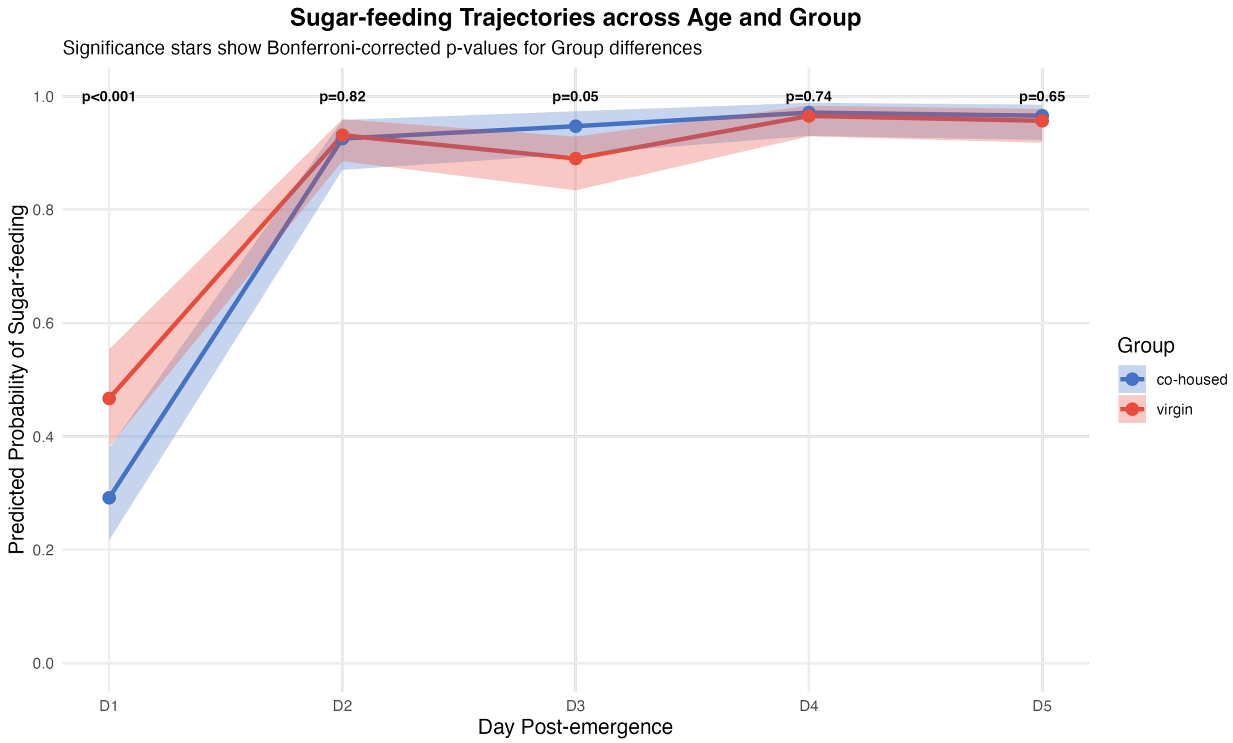

<u>Conclusions:</u> Both virgins and co-housed females show a sharp increase in their sugar-appetite from D1 to D2, followed by stabilization through D5.

Sugar-feeding behaviours are largely comparable between groups, across days. Virgin females demonstrated significantly higher initial feeding probability compared to co-housed females (D1). This difference was eliminated by D2 and remained non-significant thereafter.

Model 5: Effect of mating on the sugar-feeding behaviour of co-housed females

Random variables: BioID (replicate identity)

Fixed variables: Day(age), Mating status (yes or no)

```
model5 <- glmer(Sugar.fed_binary ~ Day * Mating + (1|BioID),
            data = mated_only,
            family = binomial)
```

Model summary:

Characteristic	Odds	Probability (%)	OR[1]	95% CI	p-value
**Day**					
D1	0.42	29.6	—	—	
D2	7.75	88.6	18.4***	8.19, 41.5	**<0.001**
D3	2.98	74.9	7.08***	2.64, 19.0	**<0.001**
D4	6.48	86.6	15.4***	4.32, 55.1	**<0.001**
D5	13511668.03	100.0	32,155,341	0.00, 458,391,919,179,031,447,577,326,110,191,003,156,070,149,023,901,469,250,371,113,818,366,346,793,494,511,616	0.841
**Mating**					
n			—	—	
y	1.70	63.0	4.05	0.92, 17.9	0.065
**Day * Mating**					
D2 * y	0.28	21.9	0.67	0.10, 4.44	0.682
D3 * y	1.76	63.8	4.18	0.52, 33.8	0.180
D4 * y	1.24	55.4	2.95	0.27, 31.7	0.373
D5 * y	0.00	0.0	0.00	0.00, 7,248,915,324,456,836,893,750,989,915,264,405,095,298,755,374,445,726,709,201,567,219,712	0.866

[1] *p<0.05; **p<0.01; ***p<0.001

Abbreviations: CI = Confidence Interval, OR = Odds Ratio

Post-hoc analysis via estimated marginal means (EM means)

Effect of mating (yes) on each day (p-value adjustment: Bonferroni method)

```
Day = D1:
 contrast estimate    SE  df z.ratio p.value
 y - n        1.40 0.757 Inf   1.846  0.0648

Day = D2:
 contrast estimate    SE  df z.ratio p.value
 y - n        1.00 0.596 Inf   1.684  0.0921

Day = D3:
 contrast estimate    SE  df z.ratio p.value
 y - n        2.83 0.754 Inf   3.753  0.0002

Day = D4:
 contrast estimate    SE  df z.ratio p.value
 y - n        2.48 0.946 Inf   2.620  0.0088

Day = D5:
 contrast estimate    SE  df z.ratio p.value
 y - n      -13.08 85.900 Inf  -0.152  0.8790

Results are given on the log odds ratio (not the response) scale.
```

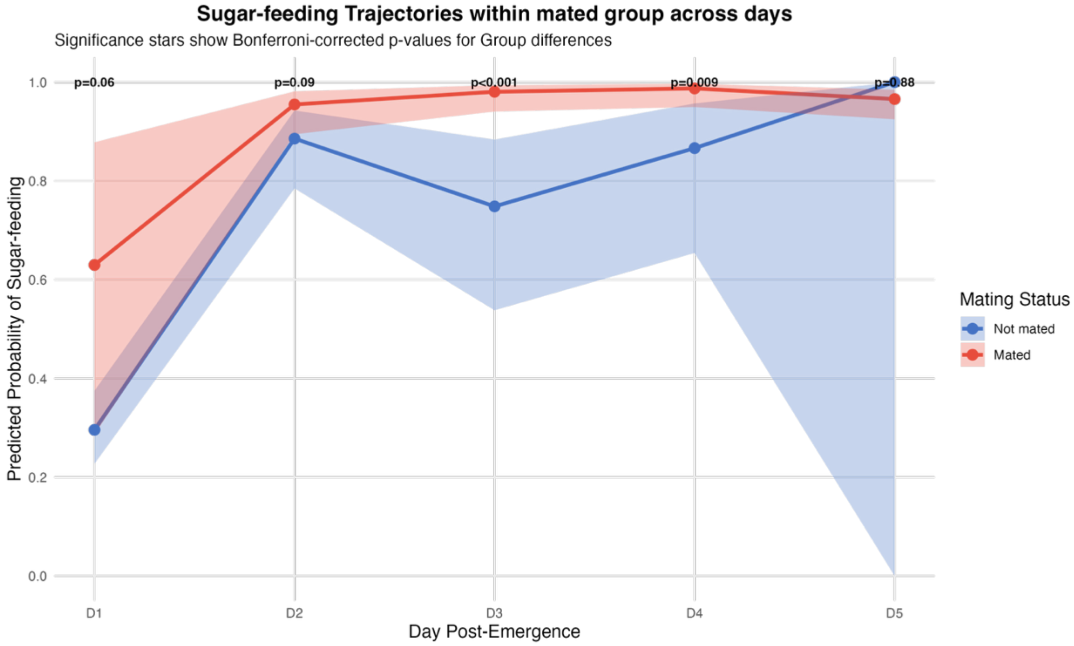

Conclusions: Mating status shows only a brief, significant effect on sugar-feeding on D3 and D4 within co-housed females: mating positively influences sugar-feeding on these days. However, it is interesting to note that no differences were observed in the feeding behaviour of virgins and co-housed females (model 4) on these days. As mating sharply increases from D2 to D4, it possibly creates maximum differences on these days within the co-housed group, which are then cancelled out when averaged together.

<u>Model 6: Whether the experimental groups differ in their sugar-feeding behaviour, post-blood meal, across age?</u>

Random variables: BioID (replicate identity)

Fixed variables: Day(age), Group (co-housed vs virgin)

```
model6 <- glmer(Sugar.fed_binary ~ Day * Group + (1|BioID),
            data = mosquito_data,
            family = binomial)
```

Model summary:

Characteristic	Odds	Probability (%)	OR[1]	95% CI	p-value
**Day**					
DB1	0.02	2.0	—	—	
DB2	0.05	4.8	3.13	0.83, 11.8	0.093
DB3	10.11	91.0	631***	180, 2,219	**<0.001**
DB4	389103395.39	100.0	24,298,538,607	0.00, 347,082,619,877,364,307,044,218,276,151,296	0.358
**Group**					
co-housed	0.02	2.0	—	—	
virgin	0.22	18.0	13.6***	4.12, 45.2	**<0.001**
**Day * Group**					
DB2 * virgin	0.12	10.7	7.21**	1.74, 29.8	**0.006**
DB3 * virgin	0.01	1.0	0.43	0.08, 2.17	0.306
DB4 * virgin	0.00	0.0	0.00	0.00, 124,369,836,581,060	0.476

[1] *p<0.05; **p<0.01; ***p<0.001

Abbreviations: CI = Confidence Interval, OR = Odds Ratio

(note: PBM abbreviated as "B")

Post-hoc analysis via estimated marginal means (EM means)

1) **Age-effects within each group (p-value adjustment: Tukey's method)**

```
Group = co-housed:
 contrast  estimate     SE  df z.ratio p.value
 DB1 - DB2    -1.14  0.678 Inf  -1.680  0.3343
 DB1 - DB3    -6.45  0.641 Inf -10.055  <.0001
 DB1 - DB4   -23.91 26.000 Inf  -0.919  0.7948
 DB2 - DB3    -5.31  0.433 Inf -12.246  <.0001
 DB2 - DB4   -22.77 26.000 Inf  -0.875  0.8179
 DB3 - DB4   -17.47 26.000 Inf  -0.671  0.9081

Group = virgin:
 contrast  estimate     SE  df z.ratio p.value
 DB1 - DB2    -3.12  0.257 Inf -12.141  <.0001
 DB1 - DB3    -5.60  0.539 Inf -10.387  <.0001
 DB1 - DB4    -5.35  0.491 Inf -10.896  <.0001
 DB2 - DB3    -2.49  0.538 Inf  -4.623  <.0001
 DB2 - DB4    -2.24  0.489 Inf  -4.569  <.0001
 DB3 - DB4     0.25  0.679 Inf   0.368  0.9830

Results are given on the log odds ratio (not the response) scale.
P value adjustment: tukey method for comparing a family of 4 estimates
```

2) **Pair-wise comparison at each day (p-value adjustment: Bonferroni method)**

```
Day = DB1:
 contrast              estimate     SE  df z.ratio p.value
 (co-housed) - virgin     -2.61  0.611 Inf  -4.275  <.0001

Day = DB2:
 contrast              estimate     SE  df z.ratio p.value
 (co-housed) - virgin     -4.59  0.398 Inf -11.523  <.0001

Day = DB3:
 contrast              estimate     SE  df z.ratio p.value
 (co-housed) - virgin     -1.77  0.562 Inf  -3.141  0.0017

Day = DB4:
 contrast              estimate     SE  df z.ratio p.value
 (co-housed) - virgin     15.95 26.000 Inf   0.613  0.5401

Results are given on the log odds ratio (not the response) scale.
```

(note: PBM abbreviated as "B")

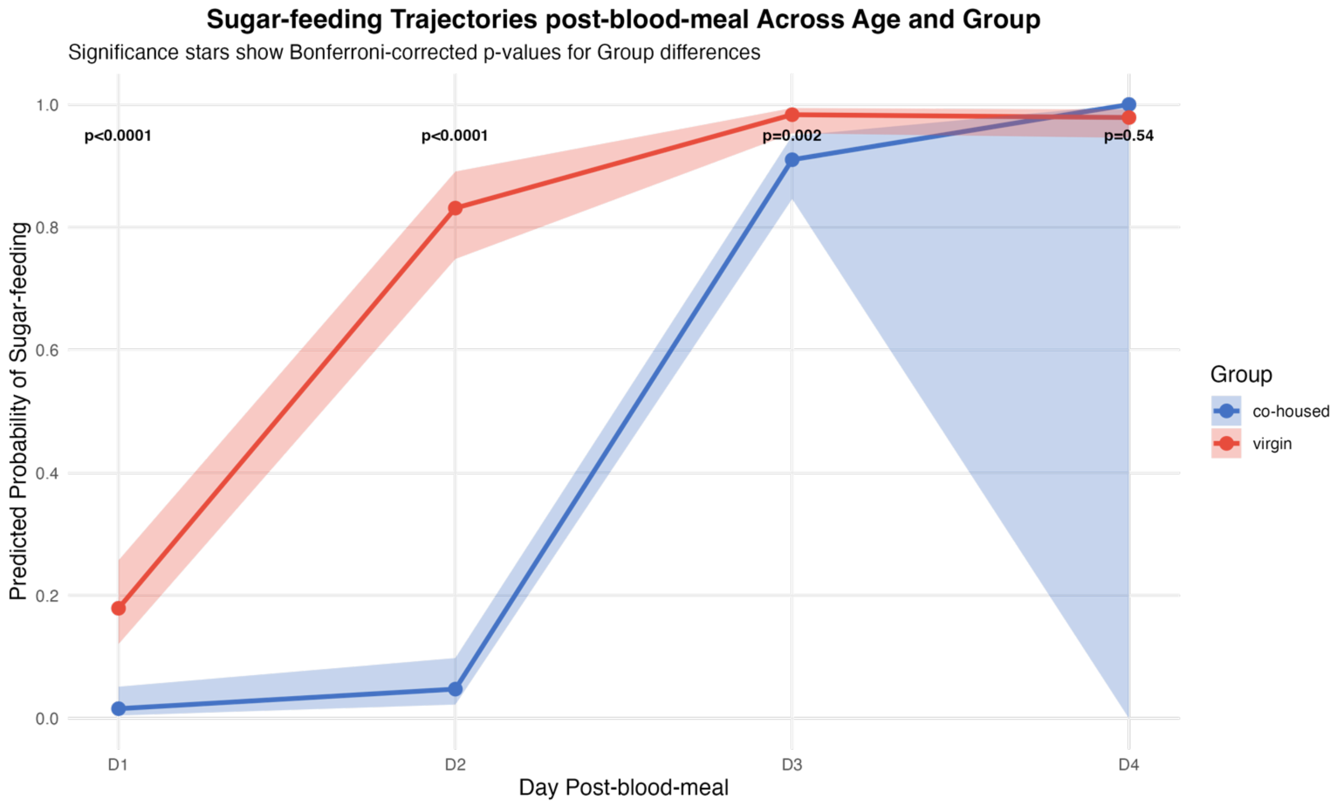

Conclusions: Both virgins and co-housed females (assumed mated) show a repressed appetite for sugar immediately after a blood meal (D1 post-blood meal). This repression is significantly stronger in mated females. Virgins start to regain their appetite by D2 post-blood meal, while the mated females continue to suppress it. By D3 post-blood meal, the appetite is restored for both groups.
